# Precise Excision of the CAG Tract from the Huntingtin Gene by Cas9 Nickases

**DOI:** 10.3389/fnins.2018.00075

**Published:** 2018-02-26

**Authors:** Magdalena Dabrowska, Wojciech Juzwa, Wlodzimierz J. Krzyzosiak, Marta Olejniczak

**Affiliations:** ^1^Department of Genome Engineering, Institute of Bioorganic Chemistry, Polish Academy of Sciences, Poznan, Poland; ^2^Department of Biotechnology and Food Microbiology, Poznan University of Life Sciences, Poznan, Poland; ^3^Department of Molecular Biomedicine, Institute of Bioorganic Chemistry, Polish Academy of Sciences, Poznan, Poland

**Keywords:** genome editing, CRISPR/Cas9, neurodegenerative diseases, repeat expansion, engineered nucleases, Huntington's disease, nonsense-mediated decay

## Abstract

Huntington's disease (HD) is a progressive autosomal dominant neurodegenerative disorder caused by the expansion of CAG repeats in the first exon of the huntingtin gene (*HTT*). The accumulation of polyglutamine-rich huntingtin proteins affects various cellular functions and causes selective degeneration of neurons in the striatum. Therapeutic strategies used to date to silence the expression of mutant *HTT* include antisense oligonucleotides, RNA interference-based approaches and, recently, genome editing with the CRISPR/Cas9 system. Here, we demonstrate that the CAG repeat tract can be precisely excised from the *HTT* gene with the use of the paired Cas9 nickase strategy. As a model, we used HD patient-derived fibroblasts with varied numbers of CAG repeats. The repeat excision inactivated the *HTT* gene and abrogated huntingtin synthesis in a CAG repeat length-independent manner. Because Cas9 nickases are known to be safe and specific, our approach provides an attractive treatment tool for HD that can be extended to other polyQ disorders.

## Introduction

Expansions of short tandem repeat sequences in functionally unrelated genes are causative factors of numerous human hereditary neurological diseases. Currently, there are nine known neurodegenerative disorders caused by the expansion of CAG repeats within the coding regions of associated genes. These disorders include Huntington's disease (HD) (Bates et al., 2015); spinocerebellar ataxia types 1, 2, 3, 6, 7, and 17 (SCA) (Paulson et al., 2017); spinal-bulbar muscular atrophy (SBMA) (Spada et al., 1991); and dentatorubral-pallidoluysian atrophy (DRPLA) (Koide et al., 1994). A positive correlation exists between the size of the expansion and the severity of symptoms, which usually appear during the 4th-5th decade of life and lead to patient's death (Duyao et al., 1993).

HD is caused by the expansion of CAG repeats in exon 1 of the *HTT* gene, which encodes huntingtin (HTT), a large protein of more than 3,000 amino acids (Saudou and Humbert, 2016). Expanded polyglutamine (polyQ) protein may form intracellular aggregates and affects numerous cellular activities inducing pathogenesis *via* a gain of toxic function. Despite many years of research on an effective treatment method, HD and other polyQ diseases are incurable, and only their symptoms can be controlled. Several different strategies have already been employed in cellular and animal models of polyQ diseases to achieve the desired therapeutic effects (Wild and Tabrizi, 2017). These strategies include the silencing of both HTT alleles in a non-allele-selective strategy and the targeting of single-nucleotide polymorphisms (SNPs) linked to repeat expansions. The repeat region itself may be targeted in an allele selective and non-selective manner (Fiszer et al., 2012; Keiser et al., 2016; Esteves et al., 2017). RNA interference and antisense oligonucleotide technologies, which have been used for many years in experimental therapy for polyQ diseases, are currently complemented with genome editing systems such as the CRISPR/Cas9 (Shin et al., 2016; Kolli et al., 2017; Merienne et al., 2017; Monteys et al., 2017; Yang et al., 2017).

Zinc finger nucleases (ZFNs) and transcription activator-like effector-based nucleases (TALENs) were the first tools that provided proof of principle for the idea of targeted inactivation of the expanded CAG repeats at a disease *loci* (Mittelman et al., 2009; Richard et al., 2014). In one of the first studies, preceding the CRISPR/Cas9 technology development, Isalan group used zinc finger proteins (ZFPs) to selectively bind and repress expanded CAG repeats in the R6/2 mouse model of HD (Garriga-Canut et al., 2012). In other approach expanded CAG repeat tracts were replaced with a normal CAG length by inducing homologous recombination in induced pluripotent stem cells (iPSCs) derived from HD patient fibroblasts (An et al., 2012). The efficiency of homologous recombination was further increased by using CRISPR/Cas9 (An et al., 2014).

The CRISPR-Cas9 system uses a small guide RNA (sgRNA) containing a 20 nt sequence complementary to the target DNA and Cas9 nuclease for site-specific cleavage of a genomic target containing a protospacer-adjacent motif (PAM) (Jinek et al., 2012). Double-strand breaks (DSBs) are repaired mainly by error-prone non-homologous end joining (NHEJ), resulting in mutations that may cause frame-shifts in open reading frames, premature translation termination and transcript degradation by nonsense-mediated decay (NMD). To increase specificity and reduce off-targeting, one of two cleavage domains in the Cas9 protein was mutated to act as a nickase (Cas9n) (Cho et al., 2014; Trevino and Zhang, 2014). Nickases generate single strand breaks (SSBs) that are repaired with high fidelity. Paired sgRNA/Cas9 nickases targeted to the opposite DNA strands enable genome editing via homology-directed repair (HDR) and have been shown to reduce off-targeting by 5- to 1.500-fold compared to wild-type Cas9 (wt Cas9) (Ran et al., 2013; Cho et al., 2014). Therefore, the paired Cas9 nickase strategy can be useful in applications that require precise genome editing such as gene and cell therapy.

To date, the CRISPR/Cas9 system has been used to selectively inactivate mutant *HTT* genes by using PAM sites generated by SNP alleles (Shin et al., 2016; Monteys et al., 2017). Although this strategy is very promising, it requires a comprehensive analysis of the *HTT* gene haplotype structure. In addition, the non-allele selective approach has been used to inactivate the *HTT* gene by using a pair of sgRNAs flanking CAG repeats and wt Cas9 in a transgenic mouse model of HD (Yang et al., 2017). Non-allele selective supression of *HTT* gene expression was achieved also by using CRISPR interference strategy (CRISPRi) in HEK293T cells (Heman-Ackah et al., 2016). In this approach nuclease null, dead Cas9 (dCas9) and sgRNAs targeting *HTT* transcription start site were used.

In this study, we examined paired Cas9 nickase strategy to inactivate the *HTT* gene by targeting sequences directly flanking the CAG repeat tract. We demonstrate that precise excision of the CAG repeats from the *HTT* gene results in the abrogation of protein synthesis in all investigated fibroblast cell lines derived from HD patients. Importantly, we also show that this specific and safe strategy leads to preservation of repeat-deficient transcript level, suggesting that the transcript may escape from NMD pathway.

## Materials and methods

### Cell culture and transfection

Fibroblasts (GM04208, 21/44 CAG in the *HTT* gene; GM04281, 17/68 CAG in the *HTT* gene; GM09197, 21/151 CAG in the *HTT* gene) were obtained from the Coriell Cell Repositories (Camden, New Jersey, USA) and grown in minimal essential medium (Lonza; Basel, Switzerland) supplemented with 10% fetal bovine serum (Sigma-Aldrich; St. Louis, MO, USA), antibiotics (Sigma-Aldrich, A5955) and non-essential amino acids (Sigma-Aldrich, M7145). HEK293T cells (16/17 CAG in the *HTT* gene) were grown in Dulbecco's modified Eagle's medium (Lonza; Basel, Switzerland) supplemented with 10% fetal bovine serum (Sigma-Aldrich), antibiotics (Sigma-Aldrich) and L-glutamine (Sigma-Aldrich). HEK293T transfections were performed using calcium phosphate method with 10 μg of plasmid DNA for 3 × 10^5^ cells (Jordan et al., 1996). Fibroblasts were electroporated with the NeonTM Transfection System (Invitrogen, Carlsbad, CA, USA). Briefly, 1 × 10^6^ to 5 × 10^5^ cells were harvested, resuspended in PBS and electroporated with 10 μg of plasmid DNA (5 μg of each plasmid from a HTT_sgRNA/Cas9n pair) in 100 μl tips using the following parameters: 1.350 V, 30 ms, 1 pulse. Fibroblasts were sorted by flow cytometry (BD Biosciences, BD FACS AriaIII) 48 h post electroporation and collected for genomic DNA, RNA and protein extraction.

### Plasmids

Guide RNA sequences for the CRISPR/Cas9 system were designed as described in Ran et al. with the use of CRISPOR software (http://crispor.tefor.net/crispor.py; Haeussler et al., 2016). Briefly, the top and bottom strand of 20-nt guide RNA were synthesized (IBB, Warsaw), annealed and ligated into the pair of FastDigest BsmBI (Thermo Fisher Scientific, Waltham, MA, USA) cut plasmids, namely, pSpCas9(BB)-2A-GFP (PX458) (Addgene, Cambridge, MA, USA) and its nickase version (D10A nickase mutant; pSpCas9n(BB)-2A-GFP (PX461)) from *S. pyogenes* (Ran et al., 2013). Ligated products were transformed into chemically competent E. coli GT116 cells (InvivoGen, San Diego, CA, USA), and the cells were plated onto ampicillin selection plates (100 μg/mL ampicillin) and incubated at 37°C overnight. Plasmid DNA was isolated using the Gene JET Plasmid Miniprep kit (Thermo Scientific) and verified with Sanger sequencing. For larger scale plasmid preparations, the Qiagen Midi kit was used (Qiagen, Hilden, Germany). The sgRNA oligonucleotide sequences are presented in Table S1.

### DNA extraction and analysis of genome editing efficiency

Genomic DNA from the HD fibroblast and HEK293T cell lines was extracted using the Cells and Tissue DNA Isolation Kit (Norgen, Biotek Corp., Schmon Pkwy, ON, Canada) according to manufacturer's instructions and quantified using a spectrophotometer/fluorometer (DeNovix, Wilmington, DE, USA). For the T7E1 mismatch analysis, genomic DNA was amplified using Phusion High-Fidelity PCR Master Mix (Thermo Fisher) with primers HD1F and HD1R spanning CAG repeats in exon 1 of the *HTT* gene. The two-step PCR amplification program was used as follows: an initial denaturation at 98°C for 3 min; 12 cycles at 98°C for 15 s, 72°C for 15 s; 21 cycles at 98°C for 15 s, 62°C for 15 s, and 72°C for 15 s; and a final elongation at 72°C for 5 min. PCR products were purified using the GeneJET PCR Purification Kit (Thermo Fisher). 400 ng of the purified PCR product was used in an annealing reaction and enzymatic digestion with the T7E1 enzyme (New England Biolabs, Ipswich, MA, USA). Cleavage products were separated in 1.3% agarose gels and detected using G-BOX. Band intensities were analyzed with GelPro software (Media Cybernetics, Rockville, MD, USA). Indel occurrence was estimated with an analysis of signal loss from the main PCR products. Briefly, the main band intensities from HTT_sgRNA-treated samples were compared to the same bands from control samples treated with the empty plasmid. Genes selected for off-target analysis were PCR-amplified with specific primer pairs (Table S1) using Phusion High-Fidelity PCR Master Mix (Thermo Fisher) and the following program: an initial denaturation at 95°C for 3 min; 30 cycles at 95°C for 15 s, 62°C for 15 s, and 72°C for 15 s; and a final elongation at 72°C for 5 min. T7E1 analysis was performed as described for the *HTT* gene.

### RNA isolation and reverse transcription-polymerase chain reaction

Total RNA was isolated from fibroblast cells using the TRI Reagent (BioShop; Burlington, Canada) according to the manufacturer's instructions. The RNA concentration was measured using spectrophotometer (DeNovix). A total of 700 ng of RNA was reverse transcribed at 55°C using Superscript III (Life Technologies) and random hexamer primers (Promega; Madison, WI, USA). The quality of the reverse transcription (RT) reaction was assessed through polymerase chain reaction (PCR) amplification of the GAPDH gene. Complementary DNA (cDNA) was used for quantitative polymerase chain reaction (qPCR) using SsoAdvanced™ Universal SYBR® Green Supermix (BIO-RAD, Hercules, CA, USA) with denaturation at 95°C for 30 s followed by 40 cycles of denaturation at 95°C for 15 s and annealing at 60°C for 30 s. The melt curve protocol was subsequently performed for 5 s at 65°C followed by 5 s increments at 0.5°C from 65°C to 95°C with *HTT-* or *GAPDH*-specific primers (sequences are listed in Table S1) on the CFX Connect™ Real-Time PCR Detection System (BIO-RAD). In order to avoid generation of two PCR products with different number of CAG repeats (two alleles of HD), primers used in qRT-PCR (HD_F and HD_R) were design to cover the HTT region downstream the CAG repeat tract. Data preprocessing and normalization were performed using BIO-RAD CFX Manager software (BIO-RAD). To confirm that the HTT transcript from the Cas9n-treated fibroblasts did not contain CAG repeats, cDNA was amplified with cDNAF and cDNAR primers flanking the repeat tract.

### Western blot analysis

A total of 30 μg of protein was resolved on a Tris-acetate SDS-polyacrylamide gel (3–8%, NuPAGE^TM^, Invitrogen, Carlsbad, CA, USA) in Tris-Acetate SDS Running buffer (Novex, Carlsbad, CA, USA) at 170 V at 4°C. After electrophoresis, the proteins were wet-transferred overnight to a nitrocellulose membrane (Sigma–Aldrich). The primary antibodies, namely, anti-huntingtin (1:1000, MAB2166, Millipore, Burlington, MA, USA) and anti-plectin (1:1000, ab83497, Abcam, Cambridge, UK), and the secondary antibodies, namely, the anti-mouse HRP conjugate (1:2000, A9917, Sigma–Aldrich) and anti-rabbit HRP conjugate (1:2000, 711-035-152, Jackson ImmunoResearch, West Grove, PA, USA) were used in a TBS/0.1% Tween-20 buffer containing 2.5% non-fat milk. The immunoreaction was detected using Western Bright Quantum HRP Substrate (Advansta, Menlo Park, CA, USA). The protein bands were scanned directly from the membrane using a camera and quantified using Gel-Pro Analyzer (Media Cybernetics).

### Sanger sequencing

DNA obtained from cell cultures transfected with plasmids was sequenced using a forward primer (HD1F). PCR products from DNA treated with Cas9 nickase pairs were separated in 1% agarose gel. Bands were extracted using the GeneJET Gel Extraction Kit (Thermo Scientific) and sequenced with the same HD1F primer.

### Statistical analysis

Statistical analysis was performed using GraphPad Prism v. 5.0 software. Data were analyzed using one-way ANOVA followed by Bonferroni's *post hoc* test (^***^P < 0.0001) with an arbitrary value of 1 assigned to the cells treated with the empty control plasmid.

## Results

### Pre-screening of HTT_sgRNA activity in HEK293T cells

**Figure 1:**
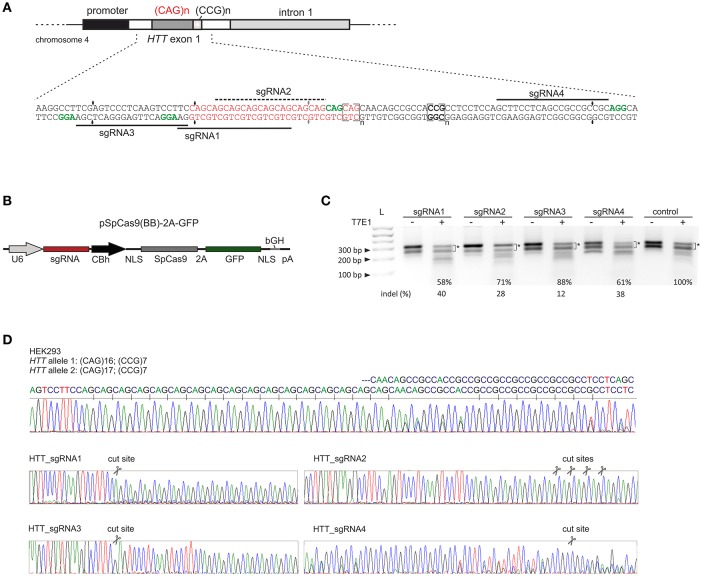
Pre-screening of HTT_sgRNAs activity in HEK293T cells. **(A)** Polymorphic CAG repeats and the following CCG repeats are located in exon 1 of the *HTT* gene. sgRNA1, sgRNA3 and sgRNA4 were designed to target the 5′- and 3′-repeat flanking sequences, respectively. Appropriate PAM sequences are highlighted in green. SgRNA2 uses a non-canonical NAG PAM sequence and targets CAG repeats directly. **(B)** Illustration depicting the CRISPR/Cas9 expression plasmid used to co-express the SpCas9 protein together with the GFP reporter marker and sgRNA under the U6 promoter. NLS, nuclear localization signal. **(C)** Analysis of HTT_sgRNA and wt Cas9 activity in HEK293T cells by T7E1 mismatch assays. The signal intensities of the two main bands (marked with an asterisk) was measured. The loss of this signal in relation to the control (100%) was used to calculate the indel frequency (%) in the samples treated with T7E1 (+). The length of the main PCR product is ~ 305 bp. Additional, faster migrating bands in samples non treated with T7E1 enzyme are secondary structure forms of the main product and their contribution is significantly reduced after denaturation of a sample directly before gel electrophoresis (see Figure S1). **(D)** Sanger sequencing of the *HTT* gene fragment included the CAG repeat tract. HEK293T cells are heterozygous in this locus with two alleles containing 16 and 17 CAG repeats. After *HTT* gene editing with CRISPR/Cas9, the sequence trace after the break site comprised a mixture of signals derived from the unmodified and modified DNA.

We designed 3 sgRNAs using *S. pyogenes* PAM sequences (NGG) located within the sequences flanking the CAG repeat tract in the *HTT* gene (HTT_sgRNA1, HTT_sgRNA3 and HTT_sgRNA4) and one sgRNA (HTT_sgRNA2) directly targeting the CAG repeats (Figure 1A). HTT_sgRNA2 was designed to use a non-canonical NAG PAM sequence that is known to be recognized by *S. pyogenes* Cas9 (Hsu et al., 2013; Zhang et al., 2014; Leenay et al., 2016). The first screening of the sgRNA activities was performed in easy-to-transfect HEK293T cells. The cells were transfected with plasmids expressing both the wt Cas9 protein and HTT_sgRNA (Figure 1B). The transfection efficiency, expressed as GFP-positive cells, was ~80% (data not shown), and genomic DNA was isolated 48 h post-transfection from unsorted cells. PCR amplification of the *HTT* gene region, including the CAG repeats, and subsequent T7E1 analysis resulted in the generation of multiple bands in both the treated and untreated control cells (Figure 1C). The HEK293T cells contain 16 and 17 CAG repeats in the two alleles of the *HTT* gene (Figure 1D). In addition, PCR products containing long stretches of repeated sequences may form various secondary structures (e.g., hairpins) in non-denaturing conditions (agarose gel electrophoresis) (Figure S1). Therefore, a determination of the exact indels frequency in this polymorphic, highly repetitive gene region was difficult using methods based on heteroduplex recognition by nucleases. However, Sanger sequencing (Figure 1D) and T7E1 analysis of the PCR products from a pool of transfected and non-transfected cells revealed that HTT_sgRNA1, HTT_sgRNA4 and HTT_sgRNA2 efficiently edited the *HTT* gene (~40, 38, and ~28% of indels, respectively) whereas HTT_sgRNA3 was the least active (~12%) (Figure 1C). This result was consistent with the fact that the sequence composition of sgRNA (GC:AT content) may influence the editing efficiency (Moreno-Mateos MA 2015). The GC content for HTT_sgRNAs ranges from 57% for the least active HTT_sgRNA3 to 81% for HTT_sgRNA4.

**Figure 2:**
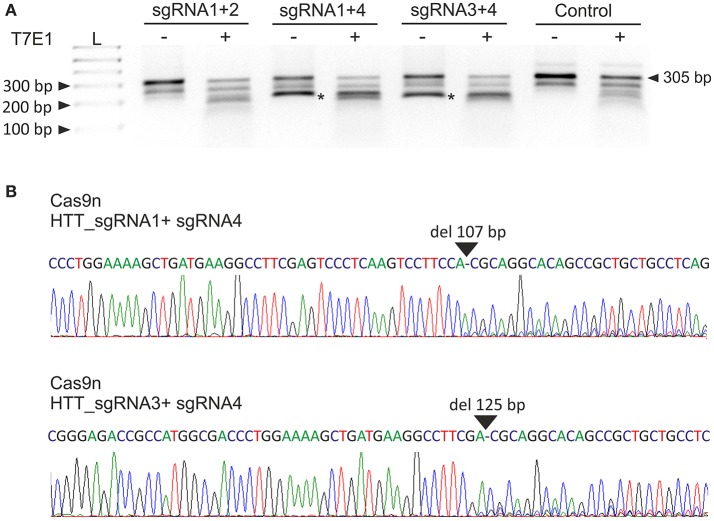
Excision of the CAG repeats from the HTT gene with paired Cas9 nickases. **(A)** DNA from HEK293T cells treated with Cas9 nickase and the HTT_sgRNA pairs was PCR amplified and subjected to T7E1 analysis. The length of the unmodified PCR fragment is 305 bp, whereas the edited products are shortened to < 200 bp (marked with an asterisk). **(B)** A representative Sanger sequencing electropherogram showing the deletion of 107 and 125 bp in HTT_sgRNA1+4- and HTT_sgRNA1+3-treated cells, respectively (marked with an arrow).

Next, we analyzed the activities of the HTT_sgRNA pairs with the Cas9 nickase protein. HEK293T cells were transfected with a pair of plasmids encoding HTT_sgRNA1 and HTT_sgRNA2, HTT_sgRNA1 and HTT_sgRNA4, and HTT_sgRNA3 and HTT_sgRNA4. T7E1 analysis (Figure 2A) and Sanger sequencing (Figure 2B) confirmed that except for HTT_sgRNA1+2, the sgRNAs functioned in pairs and generated shorter bands corresponding to HTT amplicons with 107-bp and 125-bp deletions for HTT_sgRNA1+4 and HTT_sgRNA3+4 pairs, respectively. As a result of the CAG repeat excision and frameshift mutation premature-translation-termination codon, PTC (TGA) was generated in the 3' region of *HTT* exon 1 (e.g., at the 44th codon of the 50-codon exon 1 for Cas9n_sgRNA1+4-treated cells) (Figure S2).

### Analysis of paired HTT_sgRNA/Cas9n activity in patient-derived fibroblasts

**Figure 3:**
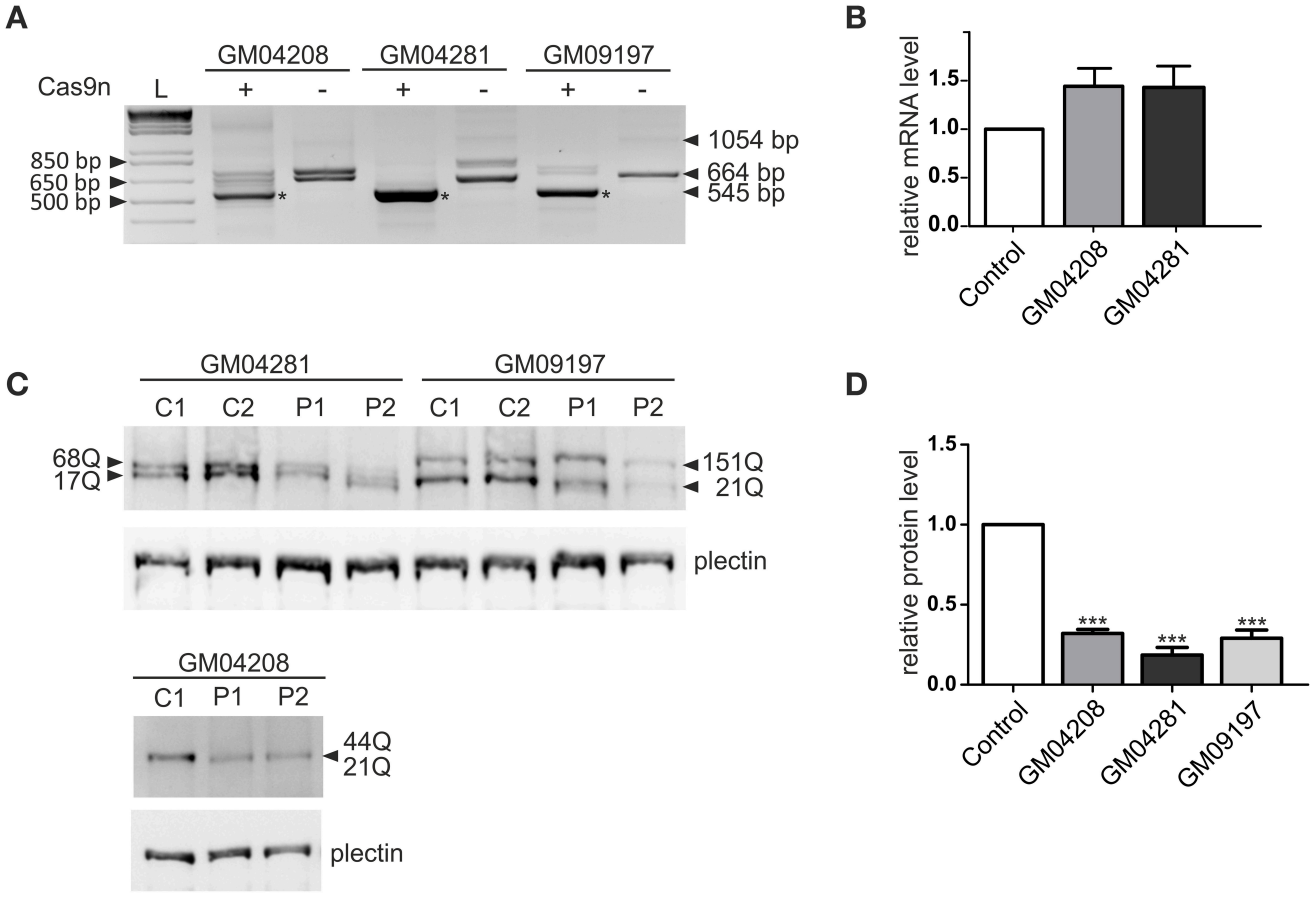
Huntingtin inactivation via the Cas9 nickase pair in patient-derived fibroblasts. **(A)** RT-PCR analysis of the Cas9n/HTT_sgRNA1+4**-**edited (+) and non-edited (–) *HTT* gene product in the three human HD cell lines containing 21/44 (GM04208), 17/68 (GM04281) and 21/151 (GM09197) CAG repeats. As a result of repeat excision, shorter PCR products (~545 bp, marked with an asterisk) are present. **(B)** RT-qPCR analysis of the HTT mRNA levels in the human fibroblast cell lines transfected with the Cas9n/HTT_sgRNA1 and sgRNA4 plasmid pairs. All samples are normalized to human GAPDH, and the results are the mean (± SEM) relative to the cells transfected with the control Cas9n plasmid (one-way ANOVA followed by Bonferroni's *post hoc* test; the difference was non-significant). **(C)** Representative western blots showing the HTT protein levels in the control fibroblasts treated with the empty Cas9n plasmids, expressing only Cas9 protein and not sgRNA (C1 and C2) and in the Cas9n/HTT_sgRNA1+4-treated cells (P1 and P2). Plectin was used as the loading control. The lengths of the polyQ tracts in both alleles of the HTT protein in each cell line are marked with an arrow. **(D)** The quantification of the huntingtin levels in the three human HD cell lines transfected with the Cas9n/HTT_sgRNA1+4 plasmid pair relative to the plectin levels. The results indicate the mean (± SEM) relative to the cells transfected with the control Cas9n plasmid (*n* = 3; one way ANOVA followed by Bonferroni's *post hoc* test; ^***^p < 0.0001).

The most active pair of HTT_sgRNAs (sgRNA1 and sgRNA4) was electroporated into HD fibroblasts containing varied lengths of the CAG repeat tract: 21/44 CAGs, 17/68 CAGs and 21/151 CAGs. GFP-positive cells were sorted by FACS before DNA, RNA and protein isolation. We confirmed by PCR and Sanger sequencing of the PCR products that Cas9 nickase with the HTT_sgRNA1+4 pair efficiently excised the targeted region of *HTT* exon 1 in the patient-derived cell lines (Figure S3). The lengths of the excised DNA fragments were between 119 and 188 bp for both alleles of the GM04208 cell line, 107 and 260 bp for GM04281 and 119 and 509 for the GM09197 cell line. As expected, the HTT transcript also did not contain the targeted sequence, which was specifically excised from the DNA by the HTT_sgRNA1+4 Cas9 nickases (Figure 3A). A shorter, 545-bp PCR product was present in the three patients-derived cell lines; however, the editing efficiency was different, with the highest observed for the GM04281 cell line. Interestingly, the level of the HTT transcript did not change in the cells treated with paired nickases, suggesting that the transcript may have escaped the nonsense-mediated mRNA decay pathway (Figure 3B). Notably, the newly generated stop codon (UGA) was localized ~20 bp from the exon/exon junction and may have been occupied by the exon-exon junction complex (EJC) (Figure S2). Despite the presence of a shortened HTT transcript, the HTT protein level was efficiently reduced by 82% in the GM04281 cell line, 68% in the GM04208 cell line, and 71% in the GM09197 cell line (Figures 3C,D). This data accurately reflects the results of the RT-PCR analysis (Figure 3A) and indicates that the length of the CAG repeat tract does not influence the excision efficiency of Cas9n. The prematurely terminated translation product (43 amino-acid protein) was not detected with the use of the N-terminal huntingtin antibody by western blot (data not shown).

### Assessment of off-target effects

*In silico* analysis using the CRISPOR tool (Haeussler et al., 2016) predicted 13 exonic off-target sites for HTT_sgRNA1 (with score > 10.00), 18 sites for HTT_sgRNA3 and 196 for HTT_sgRNA4. Notably, more than 98% of the exonic HTT_sgRNA4 off-targets had 3 to 4 mismatches with the target sequence (Table S2). Specificity score that measures the uniqueness of a guide in the genome is low for HTT_sgRNAs, because target sequence is composed of repetitive sequences (6 for HTT_sgRNA1, 66 for HTT_sgRNA3 and 40 for HTT_sgRNA4). HTT_sgRNA2 with a non-canonical NAG PAM comprised a repeated sequence and theoretically targeted every CAG repeat tract longer than 7 units. Notably, these predictions generally applied to the wt CRISPR/Cas9 activity since Cas9 nickases cut only one DNA strand that is faithfully repaired by HDR. In addition, the maximal cleavage efficiency of paired Cas9 nickases has previously been observed at sites with the tail-to-tail orientation separated by 10–30 bp (Shen et al., 2014). Potential off-target activity for Cas9n/HTT_sgRNA1+4 pair was expected to be rare as similar sequences were unlikely to occur close together elsewhere in the genome. Nonetheless, the 4 selected off-target regions for each HTT_sgRNA were PCR-amplified and analyzed with T7E1 assays (Figure S4). The *TEX13A* and *ZFHX3* genes and *TJP2* and *FBXW7* genes were tested for HTT_sgRNA1 and HTT_sgRNA4, respectively. We used DNA from HEK293T cells treated with plasmids expressing the HTT_sgRNA1+4 Cas9 nickase pair and wt Cas9/sgRNAs. Non-specific activity of the CRISPR/Cas9 system was not detected in any of the tested off-target sites.

## Discussion

To date, multiple therapeutic approaches have been described for the treatment of HD and other polyQ diseases' however, these approaches suffer from specific limitations that hinder their introduction to the clinic (reviewed in Keiser et al., 2016; Esteves et al., 2017; Wild and Tabrizi, 2017). In addition, the role of huntingtin in cell physiology and pathology is not fully understood (Saudou and Humbert, 2016), and therefore, strategies using selective silencing of the mutant allele alone and non-allele-selective silencing of both alleles are being developed in parallel. It has been shown using RNAi and antisense oligonucleotides that the knockdown of huntingtin, either the mutant or both mutant and normal is beneficial in mouse models of HD (Harper et al., 2005; Boudreau et al., 2009; Kordasiewicz et al., 2012). Recently, the CRISPR/Cas9 system was used to permanently inactivate the *HTT* gene, by using a pair of sgRNAs flanking the CAG/CTG repeats in a transgenic mouse model of HD (HD140Q knock-in) (Yang et al., 2017). Stereotactic injection of AAVs expressing sgRNAs and SpCas9 into the striata of adult mice resulted in the depletion of huntingtin aggregates in the brain, thereby alleviating motor deficits and neuropathological symptoms.

In our study, we present another repeat-depletion strategy to inactivate the *HTT* gene in which we further improve the approach by using a nickase version of Cas9 that is known to be more specific and safe than the wt Cas9. The efficiency of paired Cas9 nickase editing depends on the activity of two sgRNAs and the length of the target sequence between the two sgRNAs (Mali et al., 2013; Ran et al., 2013). We demonstrate that the pair of HTT_sgRNA/Cas9n is able to efficiently and specifically excise the repeat-containing fragment of exon 1 in three HD patient-derived cell lines differing in CAG repeat length. We show that the CAG repeat length did not influence the cutting efficiency and specificity, and the HTT protein level is reduced by ~70% in all tested models. Notably, in the case of the GM09197 cell line containing 151 CAG repeats in the mutant allele, the HTT_sgRNAs were separated by nearly 500 bp. We confirmed the specificity and safety of the paired nickase strategy by testing selected off-target loci with T7E1 mismatch detection assays.

The mechanism of this precise repeat excision and DNA repair (without scars), atypical for NHEJ is poorly known and needs further studies. However, it has been reported previously that DSBs generated by CRISPR/Cas9 near a long stretch of CTG/CAG repeats in myotonic dystrophy type 1 (DM1) locus can induce deletion of the entire repeat region (Van Agtmaal et al., 2017). Even single DSB in the region flanking the repeated sequence was sufficient to generate clean loss of repeats. Contraction of CAG/CTG repeats was also observed for ZFN and TALEN—treated human and yeast cells (Mittelman et al., 2009; Richard et al., 2014).

Interestingly, in our study the level of the shortened HTT transcript did not change, suggesting that the transcript may be NMD-resistant. The *HTT* gene contains 67 exons and has three isoforms of mRNA transcripts (Romo et al., 2017); the two predominant forms are 10,366 and of 13,711 bp (Lin et al., 1993). The longer transcript differs by an additional 3′ UTR sequence of 3,360 bp that affects mRNA localization, stability, and translation (Di Giammartino et al., 2011). A previous report showed that targeting the HTT exon 1-intron junction with CRISPR/Cas9 reduced the mRNA level by ~50% in BM-MSCs derived from the YAC128 mouse model (Kolli et al., 2017). In another study, a large deletion of approximately 44 kb of DNA using wt Cas9 and a pair of sgRNAs targeting the upstream promoter region and intron 3 resulted in the complete abrogation of mRNA and HTT protein synthesis (Shin et al., 2016). HTT mRNA resistance to NMD, observed in our study, may result from the specific localization of CRISPR/Cas9n-generated PTC in exon 1 of the multi-exonic *HTT* gene. In addition, a UGA stop codon is localized to position occupied by the EJC (~20–24 nt upstream of the exon/exon junction), which serves to orient the NMD machinery and may be masked during the “pioneer round” of translation (Popp and Maquat, 2016). A previous report showed that β-globin transcript containing PTCs in exon 1 of three-exonic gene is NMD–resistant; however, the influence of the nonsense codon localization within transcripts needs to be clarified (Thermann et al., 1998; Inácios et al., 2004; Peixeiro et al., 2011).

Genome editing with the use of a more universal CAG repeat-targeting strategy is still challenging due to the lack of specific PAM recognized by targeted nucleases, off-targeting induced by sgRNA comprising repeats and problems with the selective inactivation of mutant alleles alone. Similar problems have already been overcome by antisense and RNAi technologies (Hu et al., 2009; Yu et al., 2012; Fiszer et al., 2013). In our study CAG repeat targeting with Cas9n and HTT_sgRNA pair composed of sgRNA2 (non-canonical NAG PAM) and sgRNA1 was ineffective. However, the in-frame shortening of the CAG repeat tract with the use of genome editing tools would be the most desired and universal approach and is our goal for future studies.

## Author contributions

MO and MD contributed to the study design. MD, MO, and WJ performed experiments. MO, MD, and WK contributed to the data analysis, writing, and editing of the manuscript.

### Conflict of interest statement

The authors declare that the research was conducted in the absence of any commercial or financial relationships that could be construed as a potential conflict of interest.
