# Supplementary material for: Precise Excision of the CAG Tract from the Huntingtin Gene by Cas9 Nickases
Source: Front Neurosci. 2018 Feb 26;12:75. doi: 10.3389/fnins.2018.00075 (PMC5834764; doi:10.3389/fnins.2018.00075)
Supplement: Supplementary file 1 [file DataSheet1.pdf]

Supplementary Material

"Precise excision of the CAG tract from the Huntingtin gene by Cas9 nickases"
Dabrowska M. et al., Frontiers in Neuroscience

Supplementary Table S1. Oligonucleotides used in the study

**Oligonucleotide ID**	**Sequence (5'-3')**	**Description**
sgRNA1s	CACCGCTGCTGCTGCTGCTGCTGGA	oligo for HTT_sgRNA1 plasmid construction
sgRNA1a	AAACTCCAGCAGCAGCAGCAGCAGC	oligo for HTT_sgRNA1 plasmid construction
sgRNA2s	CACCGAGCAGCAGCAGCAGCAGCAG	oligo for HTT_sgRNA2 plasmid construction
sgRNA2a	AAACCTGCTGCTGCTGCTGCTGCTC	oligo for HTT_sgRNA2 plasmid construction
sgRNA3s	CACCGGAAGGACTTGAGGGACTCGA	oligo for HTT_sgRNA3 plasmid construction
sgRNA3a	AAACTCGAGTCCCTCAAGTCCTTCC	oligo for HTT_sgRNA3 plasmid construction
sgRNA4s	CACCGGCTTCCTCAGCCGCCGCCGC	oligo for HTT_sgRNA4 plasmid construction
sgRNA4a	AAACGCGGCGGCGGCTGAGGAAGCC	oligo for HTT_sgRNA4 plasmid construction
U6-Fwd	GAGGGCCTATTTCCCATGATTCC	Sequencing primer
HD1F	CCGCTCAGGTTCTGCTTTTA	PCR primer, sequencing primer
HD1R	GGCTGAGGCAGCAGCGGCTG	PCR primer
GAPDH_F	GAAGGTGAAGGTCGGAGTC	qRT-PCR primer
GAPDH_R	GAAGATGGTGATGGGATTTC	qRT-PCR primer
HD_F	CGACAGCGAGTCAGTGATTG	qRT-PCR primer
HD_R	ACCACTCTGGCTTCACAAGG	qRT-PCR primer
cDNAF	CCCTGGAAAAGCTGATGAAG	primer for *HTT* cDNA, sequencing primer
cDNAR	TCTTCGGGTCTCTTGCTTGT	primer for *HTT* cDNA
ZFHX3-F	CCAAATAAACCGTCCTCAGC	primer for ZFHX gene- sgRNA1 off-target
ZFHX3-R	TTCCCTTTGTGTGCCTTTTC	primer for ZFHX gene- sgRNA1 off-target
TEX13A-F	CGTCCTACCCTGCTTAGTGC	primer for TEX13A gene-sgRNA1 off-target
TEX13A-R	GGTTCGTGGTTCCAGAGAAA	primer for TEX13A gene- sgRNA1 off-target
TJP2-F	GTAGCGGCCAATTTGACAGT	primer for TJP2 gene- sgRNA4 off-target
TJP2-R	CACAAGGAGGCACTTACGC	primer for TJP2 gene- sgRNA4 off-target
FBXW7-F	CACAGAGCGAGGGAGACAG	primer for FBXW7 gene- sgRNA4 off-target
FBXW7-R	CCTCCTCAGCGTTCTCTCAC	primer for FBXW7 gene- sgRNA4 off-target

Supplementary Table S2. Predicted exonic off-targets regions for HTT_sgRNA1 and HTT_sgRNA4 (http://crispor.tefor.net)

Off-target	Guide sequence **sgRNA_1** CTGCTGCTGCTGCTGCTGGA	**PAM** AGG	Chromosome	Gene	Strand	Mismatches
1	CTGCTGCTGCTGCTGCTGG**G**	GGG	chr16	*ZFHX3*	+	1
2	CTGCTGCTGCTGCTGCTGG**G**	GGG	chr19/3'UTR	*DMPK*	-	1
3	CTGCTGCTGCTGCTGCTG**C**A	AGG	chr15	*SEMA6D*	+	1
4	CTGCTGCTGCTGCTGCTGG**C**	GGG	chr2	*APOB/ex1*	-	1
5	CTGCTGCTGCTGCTGCTGG**C**	GGG	chr1	*SDC3/ex1*	-	1
6	CTGCTGCTGCTGCTGCTGG**C**	CGG	chr4	*SDAD1*	-	1
7	CTGCTGCTGCTGCTGCTGG**C**	AGG	chr11	*AP2A2*	+	1
8	**T**TGCTGCTGCTGCTGCTGG**C**	TGG	chr22	*TCF20*	+	2
9	CTGCTGCTGCTGCTGCTGGA	GGA	chr1	*NOS1AP*	-	0
10	CTGCTG**A**TGCTGCTGCTGGA	TGA	chr2	*SOX11*	-	1
11	CTGCTG**A**TGCTGCTGCTGGA	TGA	chr2	*HDAC4*	+	1
12	CTGCTGCTG**G**TGCTGCTGGA	GGA	chrX	*TEX13A*	-	1
13	CTGCTGCTG**G**TGCTGCTGGA	GGA	chrX	*TEX13B*	-	1

Off-target	Guide sequence **sgRNA_4** GCTTCCTCAGCCGCCGCCGC	**PAM** AGG	Chromosome	Gene	Strand	Mismatches
1	**T**CTTCCTCA**T**CC**A**CCGCC**A**C	TGG	chr12	*PXN-AS1*	-	4
2	GCT**C**C**TC**CAGCCGCCGCCGC	TGG	chr8	*PLEC*	+	3
3	GCTTCC**GG**AGCCGCCGCCGC	AGG	chr16	*CDH5*	-	2
4	GCT**G**CC**G**CAGCCGCCGCCGC	AGG	chr12	*MBD6*	-	2
5	**AT**TTCCT**GG**GCCGCCGCCGC	CGG	chr14	*DIO3*	+	4
6	GC**C**TC**T**T**T**AGCC**A**CCGCCGC	CGG	chr12	*YBX3*	-	4
7	GCTTCC**C**CAGC**A**GCC**A**C**T**GC	TGG	chr17	*CDC42EP4*	+	4
8	GCT**G**CC**A**CCGCCGCCGCCGC	AGG	chr20	*DUSP15/TTLL9*	+	3
9	G**G**T**C**CCT**G**AGCCGCCGCCGC	GGG	chr17	*MMP28*	-	3
10	**T**C**C**TCCTCAGCCGCCGCC**T**C	AGG	chr12	CLEC1A	-	3
11	GCT**G**CC**G**C**C**GCCGCCGCCGC	TGA	chr4	FBXW7	-	3
12	GCT**GA**C**G**C**C**GCCGCCGCCGC	GGG	chr9	TJP2	+	4

FIGURE S1

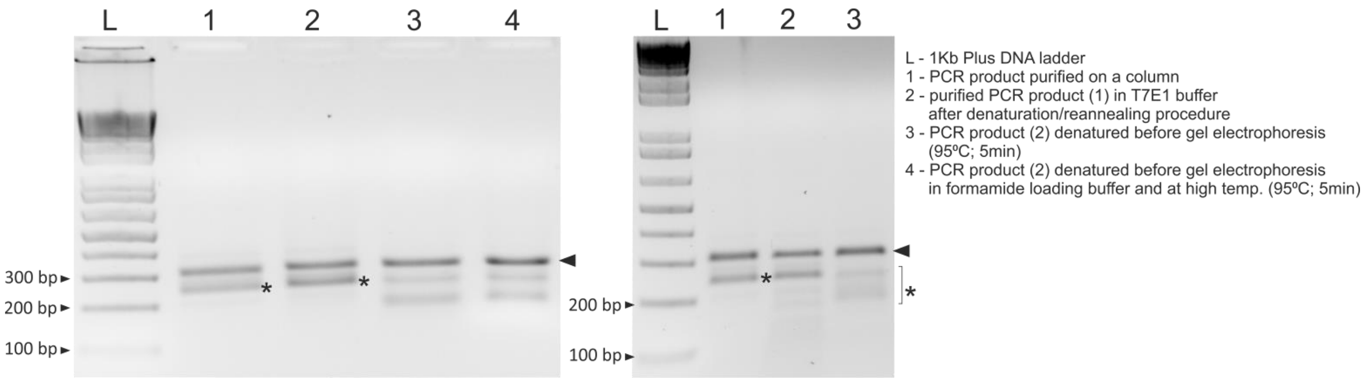

Analysis of a non-specific band generated during agarose gel electrophoresis of HTT PCR product. Genomic DNA from HEK293 cells (controls from Cas9 experiments, see Fig. 1C) was amplified using Phusion High-Fidelity PCR Master Mix with primers HD1F and HD1R spanning CAG repeats in exon 1 of the HTT gene. The two-step PCR amplification program was used as follows: an initial denaturation at 98°C for 3 min; 12 cycles at 98°C for 15 s, 72°C for 15 s; 21 cycles at 98°C for 15 s, 62°C for 15 s, and 72°C for 15 s; and a final elongation at 72°C for 5 min. PCR products were purified using the GeneJET PCR Purification Kit. 400 ng of the purified PCR product (1), PCR product after T7E1 annealing reaction (2) or PCR product after denaturation at high temperature (3) and formamide (4) were separated in 1.3% agarose gels and detected using G-BOX. Two gels represent two independent experiments. The main product (~ 305 bp) is indicated with an arrowhead. Faster migrating bands are secondary structure forms of the main product (marked with a star) and their contribution is significantly reduced after denaturation of a sample directly before gel electrophoresis.

FIGURE S2

The sequence targeted by Cas9n/HTT_sgRNAs within exon 1 of the *HTT* gene

a) Human huntingtin 3144 aa, 345kDa

ATG GCG ACC CTG GAA AAG CTG ATG AAG GCC TTC GAG TCC CTC AAG
TCC TTC **CA**G CAG CAG CAG CAG CAG CAG CAG CAG CAG CAG CAG CAG
CAG CAG CAG CAG CAG CAG CAA CAG CCG CCA CCG CCG CCG CCG CCG
CCG CCG CCT CCT CAG CTT CCT CAG CCG CCG C**CG** CAG GCA CAG CCG
CTG CTG CCT CAG CCG CAG CCG CCC CCG CCG CCG CCC CCG CCG CCA
CCC GGC CCG GCT GTG GC**T GA**G GAG CCG CTG CAC CGA CCAAAGAAAGA
ACTTTCAG...

Cas9n/HTT_sgRNA1+4; 43 aa 4,73kDa

ATG GCG ACC CTG GAA AAG CTG ATG AAG GCC TTC GAG TCC CTC AAG
TCC TTC **CAC G**CA GGC ACA GCC GCT GCT GCC TCA GCC GCA GCC GCC
CCC GCC GCC GCC CCC GCC GCC ACC CGG CCC GGC TGT GGC **TGA** GGA
GCC GCT GCA CCG ACCAAAGAAAGAACTTTCAG...

b) Human huntingtin 3144 aa, 345kDa

ATG GCG ACC CTG GAA AAG CTG ATG AAG GCC TTC **GA**G TCC CTC AAG
TCC TTC CAG CAG CAG CAG CAG CAG CAG CAG CAG CAG CAG CAG CAG
CAG CAG CAG CAG CAG CAG CAA CAG CCG CCA CCG CCG CCG CCG CCG
CCG CCG CCT CCT CAG CTT CCT CAG CCG CCG C**CG** CAG GCA CAG CCG
CTG CTG CCT CAG CCG CAG CCG CCC CCG CCG CCG CCC CCG CCG CCA
CCC GGC CCG GCT GTG GC**T GA**G GAG CCG CTG CAC CGA CCAAAGAAAGA
ACTTTCAG...

Cas9n/HTT_sgRNA3+4; 37 aa, 4kDa

ATG GCG ACC CTG GAA AAG CTG ATG AAG GCC TTC **GAC G**CA GGC ACA
GCC GCT GCT GCC TCA GCC GCA GCC GCC CCC GCC GCC GCC CCC GCC
GCC ACC CGG CCC GGC TGT GGC **TGA** GGA GCC GCT GCA CCG ACCAAAG
AAAGAACTTTCAG...

The sequence removed from exon 1 of the *HTT* gene by paired Cas9 nickases is highlighted in red and the nucleotides directly flanking the Cas9n-induced nicks are underlined and indicated in bold. As a result of the CAG repeat excision and frameshift mutation, a TGA STOP codon is generated (highlighted in yellow). The intronic sequence is indicated in blue.

FIGURE S3

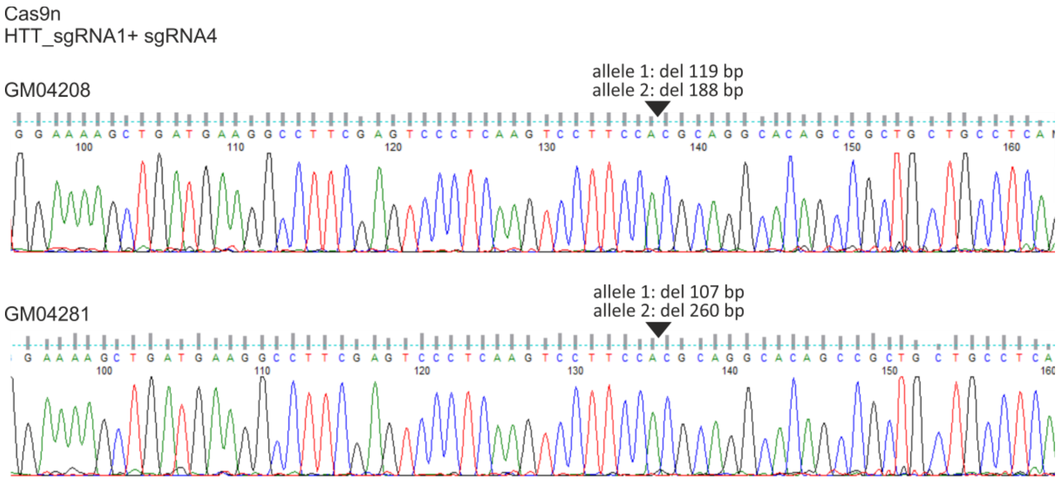

Sanger sequencing analysis of *HTT* gene editing in human fibroblast cell lines.

FIGURE S4

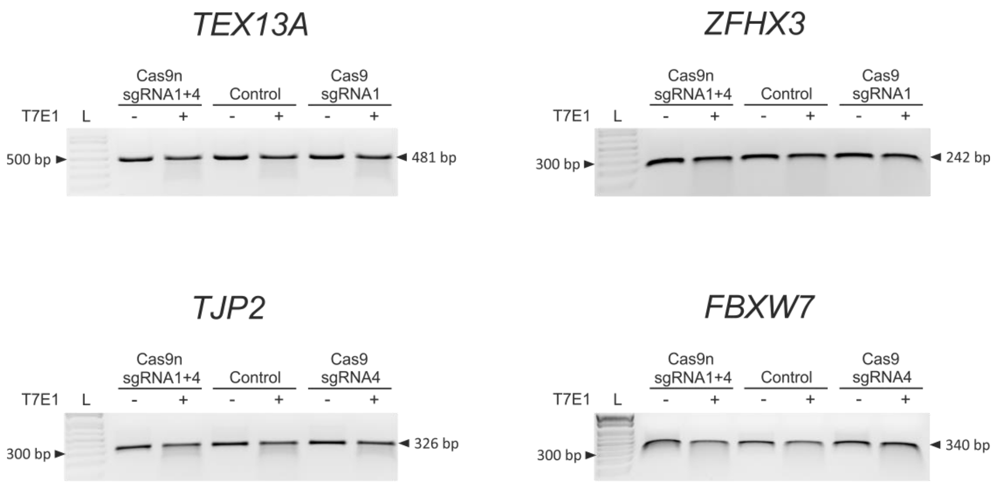

Analysis of potential off-target loci by T7E1 mismatch detection assay. DNA from HEK293T cells treated with Cas9n/HTT_sg1+4, Cas9/HTT_sgRNA1, and Cas9/HTT_sgRNA4 and from the control cells transfected with empty plasmid without sgRNAs was amplified using primers specific for HTT_sgRNA1 (TEX13A, ZFHX3) and HTT_sgRNA4 (TJP2, FBXW7) off-target genes. Next, T7E1 analysis was performed to examine cleavage activity in the potential off-target sites. Purified PCR products for appropriate genes were treated (+) and untreated (-) with the T7E1 enzyme and separated on agarose gels with a 1 kb Plus DNA ladder (L).
